# Robotic Radiosurgery for Adrenal Gland Metastases

**DOI:** 10.7759/cureus.1120

**Published:** 2017-03-26

**Authors:** Alfred Haidenberger, Sarah-Charlotta Heidorn, Nikolaus Kremer, Alexander Muacevic, Christoph Fürweger

**Affiliations:** 1 Radiation Oncologist, European CyberKnife Center Munich; 2 Medical Physicist, European CyberKnife Center Munich; 3 European CyberKnife Center Munich; 4 Chief Medical Physicist, European CyberKnife Center Munich

**Keywords:** adrenal gland metastases, cyberknife, robotic radiosurgery, radiosurgery, stereotactic body radiotherapy, radiotherapy, oligometastases, dynamic tumor tracking

## Abstract

**Introduction:**

The purpose of this study was to investigate the safety and efficacy of CyberKnife (CK) robotic radiosurgery for treatment of adrenal metastases.

**Methods:**

We performed a retrospective analysis of 23 patients with adrenal metastases who had been treated with CK between October 2006 and December 2015. Fifteen patients received chemotherapy prior to radiosurgery, all patients underwent computer tomography (CT) fluoroscopically guided percutaneous placement of one to three gold fiducials into the adrenal gland. Nineteen patients were selected for single-fraction radiosurgery with a median dose of 22 Gy, four patients were treated in three fractions with a median dose of 13.5 Gy.

**Results:**

Median follow-up time was 23.6 months. Four patients (17%) experienced local relapse during the evaluation period with a mean time of 19 months to tumor progression. The actuarial local tumor control rate was 95% after one year and 81% after two years. Three of the four patients with local recurrence were retreated with CK radiosurgery. Dynamic tumor tracking enabled accurate treatment with correlation errors less than 2 mm, despite extensive respiration-induced target motion up to 22 mm. Apart from nausea directly after treatment in five patients, we observed no early or late treatment-related side effects.

**Conclusions:**

Single fraction robotic radiosurgery for adrenal gland metastases is a safe and effective treatment option for patients who are not eligible for surgical resection.

## Introduction

The adrenal gland is a typical location for metastases of lung cancer and other primaries. They tend to occur together with multiple synchronous metastases at other sites. The common development of adrenal metastases is related to its rich sinusoidal blood supply [1]. Most patients with adrenal metastases do not show specific symptoms. Occasionally patients may have back or abdominal pain due to a large or rapidly growing tumor. In rare cases where both adrenal glands are involved, patients may develop adrenal insufficiency which includes weakness, fatigue, anorexia, nausea, vomiting, constipation, hyperpigmentation, hypotension, vitiligo, electrolyte disturbances, anemia, and eosinophilia. In severe cases, it can lead to shock and death [2]. The progress in imaging techniques has led to an increasing diagnosis of adrenal metastases which were identified during tumor staging and follow-up examinations. Positron emission tomography (PET) scanning has excellent sensitivity and specificity for distinguishing benign from malignant adrenal lesions and appears to the current investigation of choice for cancer patients with adrenal masses [3].

Metastatic cancer is commonly treated with systemic agents, optionally combined with targeted therapies. If the primary disease is well controlled and the adrenal gland is the only site of metastatic disease or if the patient is experiencing significant symptoms from a large adrenal tumor, local therapy in the form of surgery, radiotherapy, or radiofrequency ablation (RFA) is recommended. An adrenalectomy is preferred when a histological diagnosis needs to be established and the adrenal gland is the easiest site to approach [4]. For patients with metastatic lung cancer, durable long-term survival after adrenalectomy was reported for approximately 25% of cases in a meta-analysis [5].

Percutaneous, image-guided RFA is a safe and well-tolerated alternative procedure for the treatment of unresectable primary or metastatic adrenocortical carcinoma. The procedure is effective for the short-term local control of small adrenal tumors, and is most effective for tumors less than 5 cm [6].

The recent technical improvements in radiotherapy enable the radiation oncologist to use ablative doses for local treatment of adrenal metastases. High radiation doses can be delivered to the tumor with less irradiation to the surrounding normal tissue and permit selective dose escalation to the tumor [7]. This fact allows the application of larger doses over a shorter period of time, which results in a more potent radiobiological effect [2, 8-9]. Normal fractionated radiotherapy is no alternative treatment to surgical resection for the definitive management of solitary adrenal metastases because treatment responses are transient and incomplete [16-19]. Preliminary data suggest that stereotactic body radiation therapy (SBRT) is a safe and effective option in patients with adrenal metastases with equal control rates compared to surgery [2, 10].

In this retrospective study, we report about feasibility, treatment characteristics, and clinical outcome of 23 patients with adrenal gland metastases who had been treated with robotic radiosurgery from 2006 to 2015.

## Materials and methods

### Patients

Twenty-six patients treated consecutively between 10/2006 and 12/2015 were enrolled in the current study. Of these, three were lost to follow-up immediately after treatment and were therefore excluded from the analysis. Informed consent for data evaluation was obtained from all the patients.

### Treatment procedure

Prior to treatment, all patients underwent computer tomography (CT) fluoroscopically guided percutaneous placement of one to three gold fiducials into the adrenal gland. A planning CT scan was acquired with 1 mm slice thickness and overlaid with available secondary imaging (Magnetic Resonance Imaging, PET) in order to identify the 3-D target volume. Treatment planning was performed in MultiPlanTM software.

All treatments were delivered in an outpatient procedure using a CyberKnife (CK) robotic radiosurgery system [2006-2012: G4; 2013-2015: M6]. It is comprised of a compact 6 MV linear accelerator mounted on a robotic arm, a stereoscopic kV imaging system, and a camera array. Respiratory motion of the lesion is actively compensated as described previously [11]. Briefly, optical markers attached to the chest of the patient provide a continuous breathing signal that is recorded by a camera array. This signal is referenced to the tumor position as identified in multiple stereoscopic kV images of the surrogate fiducials. In this manner, a correlation model is created that is utilized by the robotic manipulator to dynamically guide a large number of non-isocentric, non-coplanar beams to the lesion while the patient is breathing freely. The correlation model is refreshed throughout the treatment by additional periodic image acquisitions.

Nineteen patients were selected for single-session radiosurgery to a median dose of 22 Gy (range 20–25 Gy) enclosing the target volume. Four patients were treated in three fractions of median 13.5 Gy (range 12–15 Gy) administered on consecutive days.

The gross tumor volume (GTV) was defined as the visible tumor structure. The GTV was enlarged by a median symmetric margin of 4 mm to create the planning target volume (PTV), resulting in PTV sizes from 4.9 to 95.9 cc. The dose was prescribed to a median isodose of 70%. Treatment characteristics are summarized in detail in Table 1.

**Table 1:** Treatment characteristics of CyberKnife for 23 patients with adrenal gland metastases

Characteristic	Number of Fractions	Range	Mean	Median
Total dose [Gy]	1	20-25	22.6	22.0
	3	36-45	40.5	40.5
D mean [Gy]	1	24.2-30.9	27.6	26.9
	3	42.4-54.5	49.0	49.6
Isodose line [%]		60-70	69.1	70
Target Vol [sqcm]		4.9-95.9	47.9	48.6

### Data analysis

Patient characteristics, treatment, and follow-up data were collected in a dedicated medical database that has been described previously [12].

Fiducial position and respiratory motion data were extracted from log files automatically stored by the CK system. This was used to calculate the respiration-induced excursion of the target during treatment as a peak-to-peak value (5-95%, to exclude outliers e.g., due to coughing) in all three translational directions for every patient. The correlation error is an important metric for treatment accuracy [13] and is defined as the difference between the actual position of the target obtained by acquiring the live x-ray images and the predicted position of the target as determined by the synchrony model. The arithmetic mean value of the correlation error was derived for each treatment.

Overall survival (OS) and local control (LC) were analyzed using STATA (v10.1 for Windows, StataCorp LP, College Station, TX, USA) and expressed as actuarial and Kaplan-Meier survival estimates. Local control was defined as tumor shrinkage or no change in size in follow-up imaging.

## Results

### Patient characteristics

The patient age ranged from 43 to 85 years with a median of 63.5 years. Sixteen right and eight left-sided adrenal gland lesions were treated, with one patient treated on both sides. Prior to radiosurgery, 15 out of 23 patients (65%) had received chemotherapy. Targets included three local recurrences after previous surgery and one after conventional fractionated radiotherapy. The demographics and clinical characteristics are summarized in Table 2.

**Table 2:** Patient demographics and characteristics HCC: Hepatocellular carcinoma; NSCLC: Non-small cell lung cancer; RCC: Renal cell carcinoma; SCLC: Small cell lung cancer.

Characteristic		
Total no. of patients = 23		
Age (years)		
mean/median min/max	61.5/63.5 43/85	
Sex	No.	[%]
Male	15	[65.2]
Female	8	[34.8]
Location		
Right adrenal gland	16	[66.7]
Left adrenal gland	8	[33.3]
Histology		
NSCLC	9	[39.1]
SCLC	2	[8.7]
RCC	7	[30.4]
HCC	1	[4.3]
Breast carcinoma	1	[4.3]
Maligne melanoma	1	[4.3]
Pancreas carcinoma	1	[4.3]
Unknown	1	[4.3]

### Target motion and treatment accuracy

**Figure 1:**
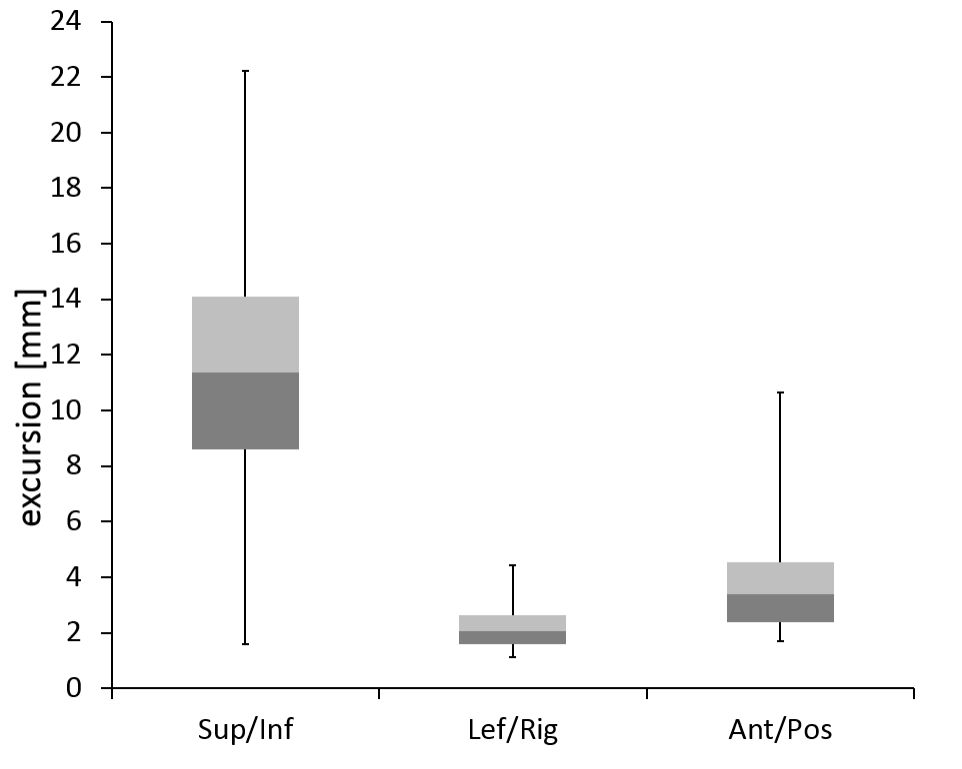
Boxplots of respiration-induced tumor excursion along the superior-inferior, left-right, and anterior-posterior axes.

All 23 patients tolerated dynamic respiration-guided delivery with CK, allowing for successful completion of every treatment. Extensive respiration-induced motion of adrenal gland targets during treatment was observed, with a median peak-to-peak value of 11.4 mm (computed as median over 24 treatments, with three translational peak-to-peak displacement values for each treatment; range 1.6–22.2 mm) along the predominant superior-inferior axis, 2.1 mm (range 1.1–2.6 mm) in anterior-posterior, and 3.4 mm (range 1.7– 10.7 mm) in left-right direction (Figure 1).

**Figure 2:**
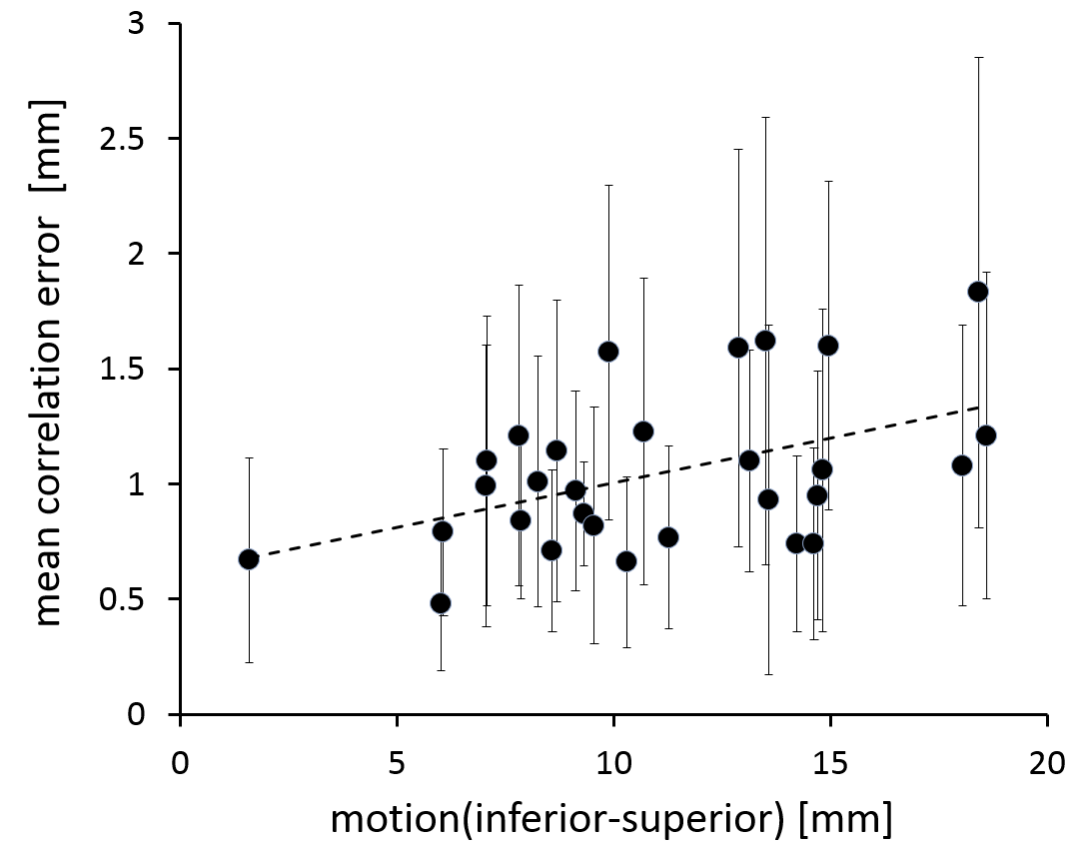
Correlation between motion along the inferior-superior axis and the mean correlation error, with one data point for each treated fraction. Error bars indicate the standard deviation of the correlation error.

In retrospective review of planning images, the tumor with minimal values of motion for all three directions was identified to be partly attached to the spine. The mean correlation error was below 2 mm for all treatments (Figure 2).

When comparing above and below median motion groups (superior-inferior), the difference in correlation error was statistically significant (Student's t-test, p = 0.025), which indicates a correlation between treatment accuracy and the extent of target motion.

### Clinical outcome

**Figure 3:**
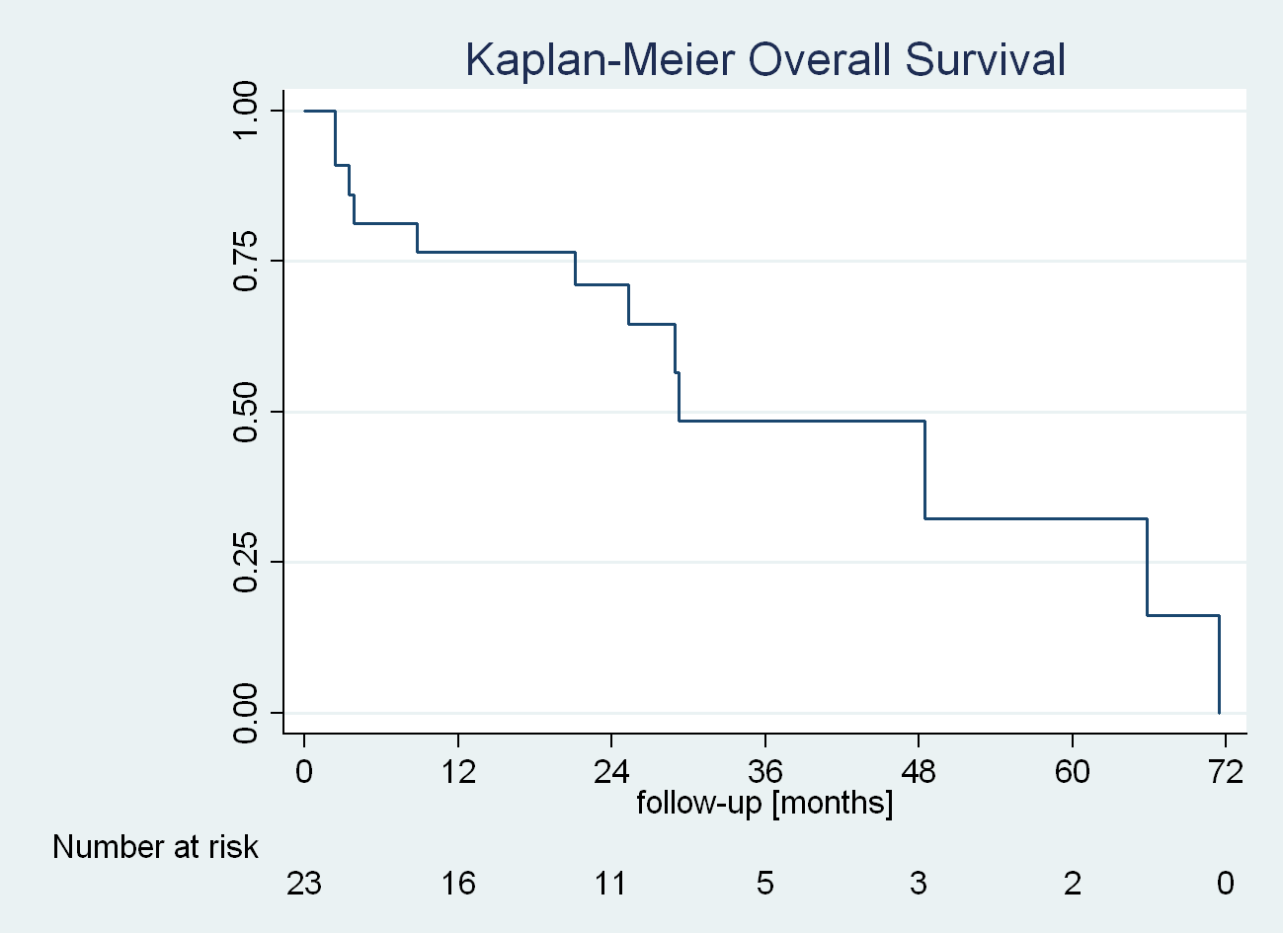
Kaplan-Meier predicted overall survival.

Median follow-up was 23.6 months (range 1.7–71.5 months, mean 25.0 months). The Kaplan-Meier predicted overall survival is shown in Figure 3.

Median overall survival after CK treatment was 29.2 months, with an actuarial survival of 77% (95% CI: 54–90%) after one year and 72% (95% CI: 47–86%) after two years.

**Figure 4:**
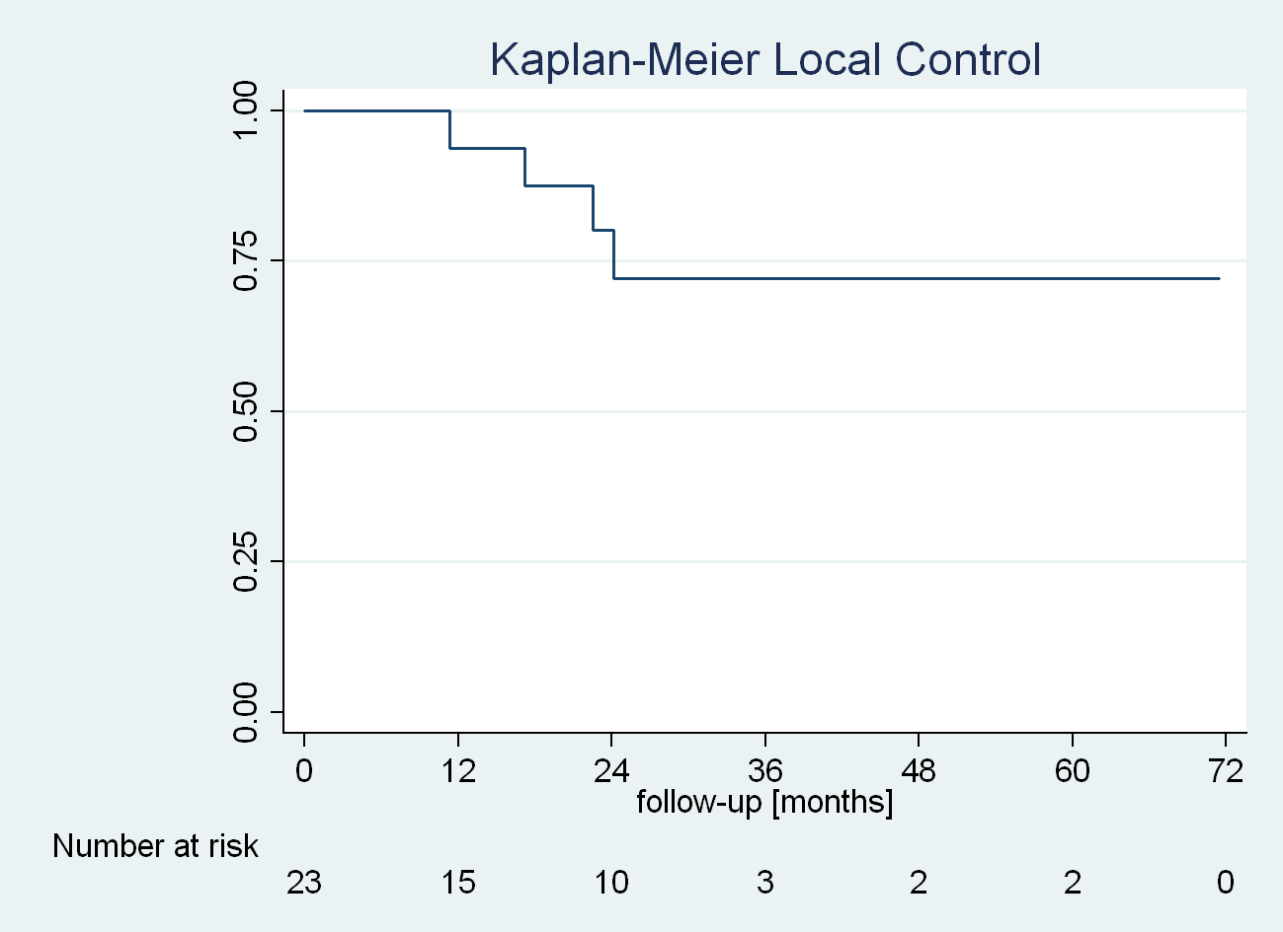
Kaplan-Meier predicted local control of the treated lesion.

The Kaplan-Meier predicted local control of the adrenal gland metastases is shown in Figure 4.

Four patients (17%) experienced local relapse during the evaluation period with a mean time of 19 months to progression, which corresponded to an actuarial local control of 95% (95% CI: 69–99%) after one year and 81% (95% CI: 51–93%) after two years. Of the four patients with local recurrence, one underwent surgery and three were retreated with CK using a single dose of 22 Gy. In these three cases, local control was retained at the time of the last follow-up. One patient developed a metastasis in the contralateral adrenal gland during follow-up, which was also treated with CK.

## Discussion

The gold standard therapy for adrenal metastases is surgical resection. Several studies reported a prolonged survival after adrenal metastasectomy [14]. Notwithstanding, many patients do have contraindications for surgery like advanced age, comorbidities or side effects after prior systemic therapy, making surgery not applicable and alternative therapies must be considered. Historically, radiotherapy has been used with palliative intent with good response rates especially in terms of pain relief [12,15]. On the basis of technical advances in recent years, it is now possible to safely apply larger doses of radiation to adrenal tumors with a limited number of fractions. The biologically equivalent doses (BED) are much higher compared with normal fractionated radiotherapy treatments. However, there are only a few studies available showing treatment results after SBRT even though it is nowadays an accepted non-invasive treatment alternative to the gold standard of surgery in selected patients [16-18]. Furthermore, the reported studies are extremely heterogeneous in terms of patient selection (primary tumors, previous treatment, performance status, disease extension) and applied dose and fractionation schedules [18]. In contrast to these studies, we here present a relatively homogeneously treated series of 23 patients harboring adrenal metastases from different primaries using high dose robotic radiosurgery.

Adrenal gland motion has been analyzed previously: Wang, et al. [19] reported motion of up to 1.2 cm in the predominant superior-inferior direction, whereas Katoh, et al. [20] observed motion of more than 2 cm in superior-inferior as well as anterior-posterior direction. Our data confirms that respiration-induced motion of the adrenal gland can indeed exceed 2 cm. For a typical 50 cc target, covering a full 2 cm trajectory would result in a more than 60% increase of tissue exposed to the prescription dose. So to minimize irradiation of adjacent healthy tissue and to accurately deliver the effective dose to the target, real-time tumor tracking is required. The large range of observed values for tumor mobility as well as correlation errors emphasizes the patient-specific nature of intrafraction motion patterns, even for targets of similar location. Still, we could demonstrate that correlation errors increase with motion of the adrenal target. This compares well to a recent analysis of liver treatments, where a weak correlation between model errors and target motion was identified [21]. As correlation errors have been recently identified as the primary random source of uncertainty for Synchrony treatments [21], and because our mean Synchrony correlation errors did not exceed 2 mm, the selected PTV margin of 4 mm seems to be reasonable.

We could achieve a one-year LC rate of 95% and a two-year LC rate of 81% with a median follow-up of 23.6 months. 19 out of 23 patients were treated in one fraction, four patients in three fractions, all patients received fiducial markers because of the extensive respiration-induced motion of adrenal gland targets during treatment. Apart from nausea directly after treatment in five patients, we observed neither relevant early nor late toxicities by following AAPM task group 101 dose constraints [22].

The one-year LC rate in the literature ranges from 44% to 100% [23-25], respectively with different applied doses and fractionation schemes. Lower doses seem to lead to lower tumor control rates as reported by Chawla, et al. [18]. Their one-year LC and OS were 55% and 44%. The prescribed dose ranged from 16 Gy (four fractions) to 50 Gy (10 fractions). No Grade II or higher toxicities were documented. In another report from Holy et al. [23], 18 patients with solitary metastases had a median progression-free survival (PFS) of 12 months and a median OS of 23 months, comparable to some of the surgical series. The applied doses ranged from 15 to 40 Gy using two to 12 fractions. Rudra, et al. [17] presented a study with 10 patients with oligometastatic disease. They reported a one-year OS of 90% with a median survival of 17.3 months. The median radiation dose was 36 Gy, the median number of fractions was three. Our analyses fit well within the data with higher BED described in the literature and showed a superior LC rate to the patient group who received lower radiation doses with lower BED. In studies with higher biological equivalent doses the local tumor control rates after two years were 70% and higher. For example, Katoh, et al. [20] treated 10 patients with SBRT using 48 Gy in eight fractions and achieved a 100% LC rate over two years. Single fraction radiosurgery (SRS) is reported in two studies with single doses of 16 to 23 Gy and LC of up to 90% after two years [24,26]. In our study, we reirradiated three patients with a local recurrence with a single dose of 22 Gy. In these three cases, local control was retained at the time of the last follow-up without additional toxicity. A similar experience is described in the study of Milano, et al. [27]. They demonstrated in their study data that even after local failure of SBRT, an additional SBRT leads to an increase in LC and OS.

## Conclusions

The present data indicates that single fractionated radiosurgery for adrenal gland metastasis is a safe and effective treatment option. Although there is only very limited information about SRS in the literature, SRS and in larger lesions hypofractionated SRS can be considered an effective alternative treatment for patients with adrenal metastases who are not eligible for surgical resection. Until treatment guidelines are more refined, we recommend to discuss every single case in the framework of an interdisciplinary team of surgeons, medical oncologists, and radiation specialists. Our initial experience must be confirmed in a study with more patients and a longer follow-up.
